# Mapping evidence on the distribution of uterine fibroids in sub-Saharan Africa: A scoping review protocol

**DOI:** 10.1371/journal.pone.0298246

**Published:** 2024-07-03

**Authors:** Vuyisile Ginindza, Makandwe Nyirenda, Mbuzeleni Hlongwa, Themba G. Ginindza

**Affiliations:** 1 Discipline of Public Health Medicine, School of Nursing and Public Health, University of KwaZulu-Natal, KwaZulu-Natal, South Africa; 2 Public health Societies and Belonging, Human Sciences Research Council, Pretoria, South Africa; 3 South African Medical Research Council, Burden of Disease Research Unit, Parowvallei, Cape Town, South Africa; 4 Cancer & Infectious Diseases Epidemiology Research Unit (CIDERU), College of Health Sciences, University of KwaZulu-Nata, KwaZulu-Nata, South Africa; Teikyo University, School of Medicine, JAPAN

## Abstract

**Background:**

Uterine fibroids are the most common pelvic benign tumours found in reproductive-aged women and may affect up to 70% of all women by menopause. Uterine fibroids place a heavy burden on women and society resulting in poor quality of life, impaired self-image, and impaired social, sexual, emotional, and physical well-being of affected individuals.

**Aim:**

This study aims to map the evidence on the burden of uterine fibroids in Sub-Saharan Africa; uterine fibroids’ burden by age, uterine fibroids’ geographic burden, uterine fibroids’ cost estimation and reported experiences among women diagnosed with uterine fibroids.

**Setting:**

Articles will be selected from countries within Sub-Saharan Africa

**Methods and analysis:**

This scoping review will be guided by the Arksey & O’Malley framework, enhanced by Levac et al (2010). The following electronic databases will be searched; PubMed, EBSCOhost (Cumulated Index to Nursing and Allied Health Literature and Health Source), Medical Literature Analysis and Retrieval System Online, Cochrane Library, Scopus, Web of Science, Africa Journal Online, and Google Scholar. The Population Concept and Context (PCC) framework will be used and the PRISMA flow diagram will also be used to show the literature search and selection of studies. Descriptive data analysis will be used; results will be presented in themes, narrative summaries, tables, and charts.

**Discussion:**

The study anticipates finding relevant literature on the distribution of uterine fibroids, the burden of uterine fibroids in terms of geographic distribution, age distribution, and cost approximation related to the disease. This will assist in identifying research gaps to guide future research contribute to the body of scientific knowledge and develop preventative strategies for the disease.

## Introduction

Uterine fibroids (UFs) (also called leiomyomas or fibromyomas) are non-cancerous growths in or on the muscular wall of the womb (the myometrium) [1]. They are classified according to the type of growth and location in the uterus and are the most common female genital tract tumours, affecting 20–25% of women, especially between 30–45 years old [2].

It has been observed that UFs have a great bearing on women’s health physically, emotionally, and psychologically, yet very few studies exist on UFs’ primary prevention and care [3]. This signifies the need to embark on further UFs research to identify gaps and explore possible solutions. Zheng Lou et al (2023), alluded to the fact that the global burden of UFs is a cause of concern in the middle and low socio-demographic quintiles, so it is important to increase UFs public awareness, increase UFs medical investments and improve UFs levels of care to reduce the burden [1]. There is no direct etiology of UFs that has been identified; however, some associated risk factors have been identified. Some are non-modifiable risk factors (age of menarche, genetics, and heredity), and others are modifiable risk factors (diet, weight, environmental contaminants, and lack of vitamin D) [4]. A recent systemic review on the epidemiology of UFs concurs with the fact that there is a lack of strong studies in SSA on the prevalence and risk factors of UFs [2]. This scoping review will search the literature not only on prevalence and risk factors but will include the reported cost burden of UFs and the experiences of women diagnosed with UF.

The burden of UFs has been linked to poor work and household productivity, leading to increased work absenteeism and household presenteeism [5]. Earlier UFs studies highlighted the psychological impact of the disease; most women with the disease experienced a lot of fear [6], while a recent systematic review indicated that, the psychosocial impact of UFs showed lower quality of life scores among women diagnosed with UFs before treatment of the disease and an improvement after treatment [3].UFs present with different signs and symptoms, including excessive vaginal bleeding, lower abdominal pains, dysmenorrhea, urinary frequency, and recurrent abortions, which result in more stress on the affected women [7]. It is evident that UFs have a great physical and psychosocial impact on affected women; fear and discouragement and altered domestic and social lives [8]. Most participants in a cross-sectional national survey amongst French women indicated that UFs negatively affected their quality of life, resulting in poor self-image due to changes in abdominal size and shape, increased fear of taking extra care due to vaginal bleeding, mood swings, loss of sexual desire, social isolation, and feeling worn out and discouraged in most instances [9]. A recent systematic review describing the causes, impact, and treatment of UFs, highlighted that sub-mucosal UFs are associated with infertility [4], while Genzalo R. et al (2020) noted that pregnant women with UFs are at high risk of preterm birth [5].UFs have also been noted to affect the psychological aspects of the affected women, resulting in feelings of fear, worry, and frustration before and after the diagnosis of UFS [11]. The burden of the UFs is extensive; it affects women holistically and cannot be ignored. The need for more research is essential. This specifies the need to raise public awareness of UFs in both urban and rural areas as early as possible in women’s lives to reduce the burden of the disease.

This protocol is part of a large research study that aims to determine the burden of uterine fibroids in the Kingdom of Eswatini among women. The main aim of this scoping review is to highlight the available literature on the burden of uterine fibroids amongst women in SSA; the reported prevalence of UFs in terms of age, geographic distribution, reported cost, and reported experiences of the women diagnosed with UFs or have done surgery related to UFs; myomectomy, hysterectomy women. The results of this study will assist in identifying literature or types of evidence in the given field, identifying research gaps, informing future research, and providing strategic and scientific information to better inform policy.

## Methodology

This scoping review will be guided by the Arksey & O’Malley (2005) framework [6] enhanced by Levac et al (2010**)** [7]) and hence the following six methodological stages will be used; i) identifying research questions, ii) identifying the relevant studies, iii) study selection, iv) charting the data v) collating, summarising and reporting the results, and vi) consultation [8]) The PRISMA-SCR guideline will be followed for reporting. This scoping review will synthesize evidence currently available in peer-reviewed sources. No human or animal participants will be recruited; therefore, ethical approval is not necessary.

### 1. Identifying research questions

**Main question**:

1. What is the evidence on the burden of UFs in Sub-Saharan Africa?

**Sub-questions**:

What are the reported geographic distributions of UFs in SSA?

What is the reported age prevalence of UFs in SSA?

What are the reported cost approximations of UFs in SSA?

What are the reported UFs experiences among women diagnosed with UFs?

The scoping review will use the Population Concept and Context (PCC) framework for the eligibility of research questions as shown in Table 1.

**Table 1:** Population concept and context framework.

Criteria	Determinants
**P-Population**	Women of all races, Women above the age of 18 years Women in Sub-Saharan Africa
**C-Concept**	UFs burden; geographic distribution, UFs prevalence, incidence, and mortality Age distribution, Cost approximation of UFs and reported experiences of women diagnosed with UFs or who have done surgery related to UFs; hysterectomy, myomectomy, laparoscopic procedure, and embolization
**C-Context**	Sub-Saharan Africa

#### Participants

All articles including women from the ages of 18 years and above, women of all races and diagnosed with uterine fibroids or had surgery related to uterine fibroids; myomectomy; laparoscopic procedures, hysterectomy.

#### Concept

The concept of interest is Uterine Fibroids; the distribution of uterine fibroids in SSA, the burden of UFs in terms of geographic distribution, age distribution, and cost approximation of UFs in SSA, and reported experiences of women diagnosed with UFs or who have done surgery related to UFs; hysterectomy, myomectomy, laparoscopic procedure and embolization.

#### Context

The articles that will be considered for inclusion in this review are those done in SSA countries.

### 2. Identifying relevant studies

The following databases will be used as information sources in searching for relevant studies; PubMed, EBSCOhost (CINAHL and Health Source), Medline, Cochrane Library, Scopus, Web of Science, Google Scholar, Africa Journal online, World Health Organization (WHO**)** library databases, and reference lists and grey literature, including conference abstracts, presentations, regulatory data, working papers and other reports to access articles that are relevant to the goal of the proposed scoping review, guided by the study inclusion and exclusion criteria show in Table 2.

**Table 2:** Shows the Inclusion criteria and exclusion criteria.

Inclusion criteria	Exclusion criteria
Studies published in English and other languages with an English version will be included in the study	Studies presenting evidence on any related disease other than UFs
Studies published between year 2000 and 2023 will be included in the study	Articles that are unavailable as full texts will be excluded
Studies presenting evidence on UFs in women above 18 years	Studies done among women from SSA but living outside SSA
Studies presenting evidence on UFs in women above 18 years	
Studies conducted in Sub-Saharan Africa	
Studies on prevalence of UFs	
Studies on reported cost approximation of UFs	
Studies on the experience of women diagnosed with UFs or who had surgery related to UFs; hysterectomy, myomectomy, embolization, and laparoscopic procedures	

The following keywords will be used in the search: uterine fibroids, leiomyoma, myomas, fibromyoma, prevalence, experiences, mortality, women, hysterectomy, myomectomy, laparoscopic procedures, and embolization only in the SSA; Congo, Nigeria, South Africa, Mauritius, Botswana, Ghana, Malawi, Angola, Tanzania, Ethiopia, Zimbabwe, Zambia, etc.

The keywords and MeSH terms will be used to search for studies, guided by the predetermined PCC format and research questions. They will be separated or joined into phrases by the Boolean terms ‘OR’ and ‘AND’. Terms such as uterus (MeSH term) OR womb, Fibroids OR Fibromas, Fibroids OR leiomyoma, fibroid (MeSH term) OR myoma, parity (MeSH term), women OR females, childbearing stage OR premenopausal stage, surgical procedures or myomectomy (MeSH term), surgical procedures or embolization (MeSH term), surgical procedures or hysterectomy (MeSH term), Prevalence, Morbidity and mortality, epidemiology and risk factors.

All Studies published between the years 2000 to 2023, published in English and any other language with an English version will be included in the study.

### 3. Study selection

**Fig 1:**
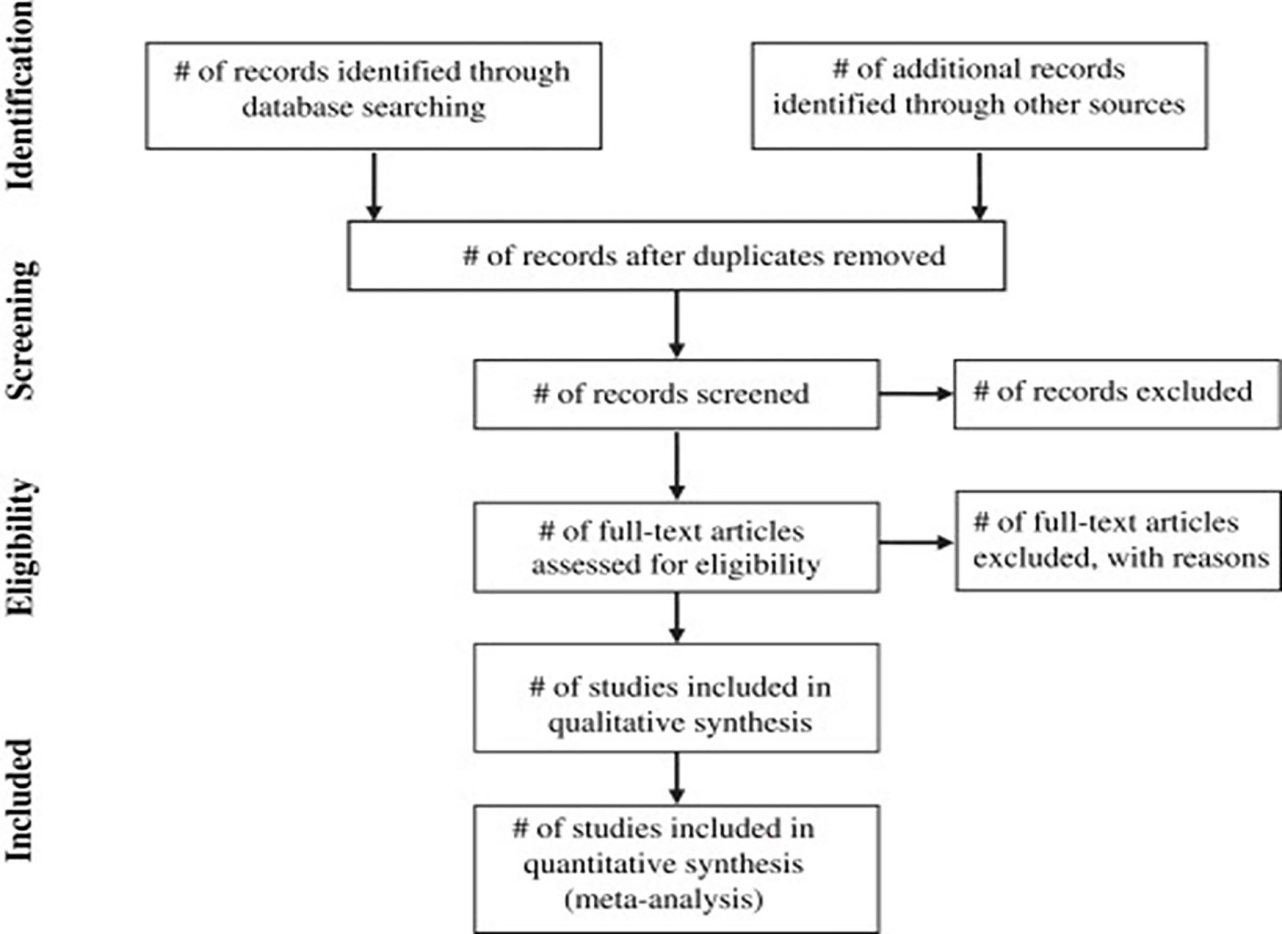
The adoped PRISMA flow diagram showing the proposed search strategy.

The study selection process will be conducted in stages; using the adopted PRISMA flow diagram shown in Fig 1 as a search strategy, aligning it to the PCC framework and the inclusion and exclusion criteria shown in Table 2 below;

#### The process of study selection

Two reviewers will be involved in this stage, the principal investigator and the librarian. First and foremost the two will meet to discuss the scoping review process for quality purposes and make amendments where needed as recommended by Levac, 2010 [7]). The study selection process will be piloted before the actual selection of the articles. A few databases will be identified; PubMed and Google Scholar where a random of 10 articles will be extracted, using the index terms; uterine fibroids, leiomyoma, myomas, and fibroma as shown in the example in Table 3.

**Table 3:** Example of a Pilot search from PUBMED.

Date	Database	keywords	Texts-type	Total
**07/01/2024**	PUB MED	Terms such as uterus (MeSH term) OR womb, Fibroids OR Fibromas, Fibroids OR leiomyoma, fibroid (MeSH term) OR myoma Uterine myoma, Sub-Saharan region,	Full texts	19

The reviewers will work independently screening the title and abstract. After this they will meet to discuss their findings to reduce any ambiguities related to the search; research questions and checking the relevancy of questions. The harmonizing meetings will be continuous in every stage of selection until the final stage and if we do not reach a consensus on certain articles, those articles will not be included in the study.

The reviewers will continue to screen full texts. At this stage, all the identified keywords, index terms, and all the identified databases will be used to search for the articles. To enhance further retrieval of all related articles, regional groupings names like Southern African Development Community (SADC), East–Africa, and Southern Africa will be used in the search. The names of the Sub-Saharan African countries; Nigeria, Malawi, Mozambique, Angola, Botswana, Chad, Ghana, Gambia, Ethiopia, Sierra Leone, Somalia, and South Africa to list a few will also be used. Fig 1, is the proposed PRISMA flow diagram that will be used to report the search, and screen the results.

The search results will be exported to an Endnote library/ the Endnote X9 referencing software which will be used and any duplicated studies will be removed. Before exportation, the reviewers will have a final deciding meeting to minimize errors. A detailed search record will be documented including search date, number of studies, source of studies, and keywords. The electronic database will be recorded in a table showing the search date, electronic database, keywords searched, number of studies retrieved, and number of selected studies as shown in Table 4 as shown below.

**Table 4:** Electronic database record.

Search date	Electronic database		Number of studies retrieved	Number of selected studies
**10/02/2024**	Google	fibroid (MeSH term) OR myoma	12	4

### 4. Charting the data

A data charting form will be developed based on the aim of the scoping review to determine which variables to extract and to rightly address the research questions. We will use both inductive and deductive reasoning to arrive at the themes. The extracted information will be as the author’s original concept, not our understanding to minimize bias. The data charting form will be developed guided by the JBI template source of evidence details, characteristics, and results extraction instrument [9]). This will be piloted interchangeably by the two independent reviewers, the principal investigator, and the librarian before actual research. A random sample of 10 of the already selected articles will be done, after which they will meet to determine the effectiveness of the tool or inconsistencies. Amendments to the form will be made as necessary. Specific information will be extracted to address the research questions; the source of study, the study aims, authors, publication dates, study design, population, country of study, area of care, significant results, and conclusion as indicated in Table 5.

**Table 5:** Data charting table.

Author	Publication year	Source	Country of origin	Area of care	Study aim	Study design	Population /context/concept	Results	Significant findings	Author’s conclusion
**M Adawe et al**	2022	Google scholar	Ghana	Gynaecology Clinic,	To determine the prevalence, clinical presentation and factors associated with uterine fibroids among women	Uterine fibroids cause significant morbidity	women	Prev- was 28.2%	risk factors included overweight and age group of 31 to 50 years	Uterine fibroids cause significant morbidity

### 5. Collating, summarising, and reporting the results

This scoping review aims to map the available literature on the distribution of uterine fibroids amongst women in SSA; approximating the burden of UFs in terms of age distribution, geographic distribution, associated cost, and mapping literature on reported experiences of the women diagnosed with UFs or have done surgery related to UFs; myomectomy, embolization, laparoscopic procedures, and hysterectomy. The data will first be summarised according to the study authors, year of publication, source, country of origin, area of care, study aim, study design, and concept.

The nature of data analysis in scoping reviews is largely determined by the purpose of the review and the reviewers’ evaluations [9]). The results from this scoping review will be described about the research question and in the context of the overall scoping review purpose. Descriptive analysis like the mean, mode, and median will be used and data will be presented in a narrative form, tables, and charts.

**Fig 2:**
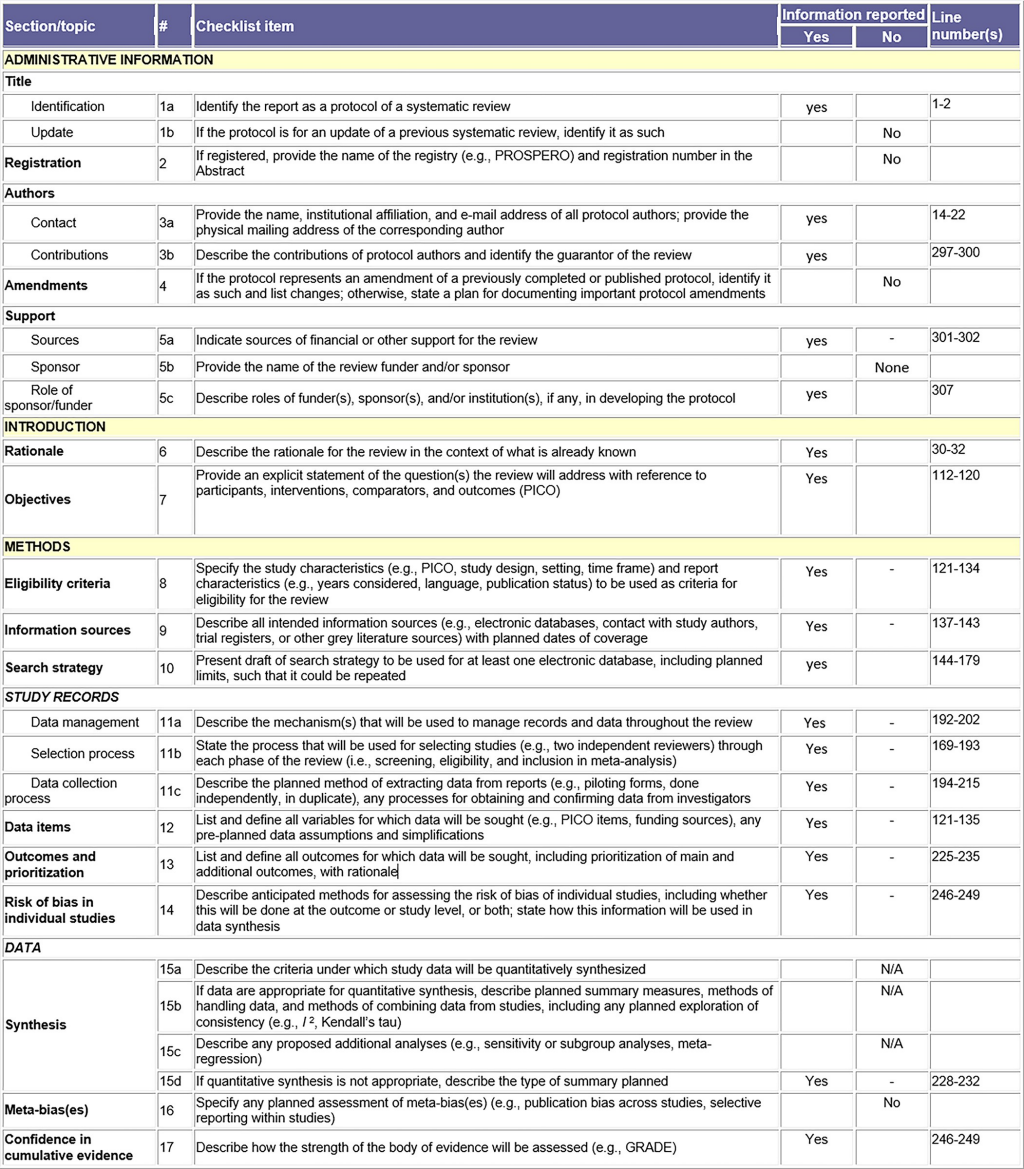
Preferred reporting items for systematic reviews and meta-analysis extension for scoping reviews (PRISMA-ScR) checklist.

The reporting of the scoping results process will follow the Preferred Reporting Items for Systematic Reviews and Meta-Analyses Extension for Scoping Reviews (PRISMA-ScR) checklist [9]) uploaded as Fig 2 in additional information.

### 6. Consultation

This stage is optional in Arksey and O’Malley’s original framework for conducting scoping reviews, whereas Levac et al recommend that this stage is an essential component of the scoping review methodology [8]). However, in this scoping review, this stage will be omitted as the focus is mapping the evidence on distribution of UFs so, there will not be any consultation of stakeholders.

#### Quality assessment

Recent research on the scoping review methodology agrees on the fact it is not obligatory to do quality assessments in scoping reviews [10,11]). The methodological quality or risk of bias of the included articles in this scoping review will not be appraised.

#### Ethical consideration

This scoping review will synthesize evidence currently available in peer-reviewed and non-peer-reviewed sources. No human or animal participants will be recruited. Therefore, ethical approval is not necessary.

#### Dissemination of results

An article reporting the results of the scoping review will be submitted for publication to a scientific journal, and presented at relevant conferences or workshops with professionals; Eswatini Sexual Reproductive Health conferences, The Eswatini United Nations Population Fund (UNFPA) related conferences, and Eswatini nurses-related workshops.

## Discussion

Uterine fibroids are a public health problem and the most common female genital tract tumours affecting 20–25% of women in genital activity especially young adults between 30–45 years [10]. Despite this, there is still a lack of epidemiological information on UFs in the SSA region. Recent UFs studies identified that UFs pose a substantial negative impact on the quality of life amongst women in SSA. Many women in Africa suffer greater morbidity and mortality from fibroid disease due to lack of knowledge, lack of access to care, late presentation, disease complications, poor management, affordability issues, and poor nutritional status.

The focus of this scoping review is to find evidence on the distribution of uterine fibroids, the burden of uterine fibroids in terms of geographic distribution, age distribution, cost approximation of UFs, and experiences of women diagnosed with UFs or who had surgery related to UFs; hysterectomy, myomectomy, embolization, and laparoscopic procedures. The results of the study will show the extent and nature of the problem of UFs in SSA, and help to answer broad questions, identify gaps on the topic, and inform the development of interventions.

### Limitations of the study

The limitation of this study is that the study findings may be broad due to the broad nature of the research question and the authors might require additional steps to synthesize and come up with relevant conclusions. Also due to the broad focus of this scoping review, the selection of databases, and the use of search terms, some studies might be missed. Another limitation could be the inclusion criteria of research articles that have an English version, this could limit access to research articles on the subject that are published in other languages. The fact that the review is targeting studies published between the years 2000 to 2023 may result in the team missing critical information on articles published earlier than the year 2000. It can be argued that since scoping reviews emphasize more on the comprehensiveness of literature rather than the quality of evidence the quality appraisal step does not apply. However, gaps in the literature related to low-quality study designs may not be ascertained.

### Conclusion

We anticipate finding relevant literature on the burden of uterine fibroids (geographic distribution, age distribution, cost approximation, and experiences reported by women diagnosed with UFs or who had any procedure reported to UFs.) in the sub-Saharan region as our primary outcomes. Research literature on uterine fibroids’ risk-associated factors will be considered as our secondary outcomes. The study findings will benefit researchers and healthcare providers in this neglected area.

## Abbreviations

UFs: Uterine fibroids
PCC: Population Concept and Context
MMAT: Mixed Method Quality Appraisal
SSA: Sub-Saharan Africa
PRISMA: Preferred Reporting Items for Systematic Review and Meta-Analysis
